# Outcome and serum ion determination up to 11 years after implantation of a cemented metal-on-metal hip prosthesis

**DOI:** 10.3109/17453670902947408

**Published:** 2009-04-01

**Authors:** Jean-Yves Lazennec, Patrick Boyer, Joel Poupon, Marc-Antoine Rousseau, Carine Roy, Philippe Ravaud, Yves Catonné

**Affiliations:** ^1^Department of Orthopaedic Surgery, Pitié-Salpétrière HospitalParisFrance; ^2^Department of Orthopaedic Surgery, Bichat HospitalParisFrance; ^3^Laboratory of Toxicology, Lariboisière HospitalParisFrance; ^4^Department of Statistics, Bichat Hospital, Université Paris Diderot, Asistance Publique-Hopitaux de ParisParisFrance

## Abstract

**Background and purpose** Little is known about the long-term outcome of cemented metal-on-metal hip arthroplasties. We evaluated a consecutive series of metal-on-metal polyethylene-backed cemented hip arthroplasties implanted in patients under 60 years of age.

**Methods** 109 patients (134 joint replacements) were followed prospectively for mean 9 (7–11) years. The evaluation included clinical score, radiographic assessment, and blood sampling for ion level determination.

**Results** At the final review, 12 hips had been revised, mainly because of aseptic loosening of the socket. Using revision for aseptic loosening as the endpoint, the survival rate at 9 years was 91% for the cup and 99% for the stem. In addition, 35 hips showed radiolucent lines at the bone-cement interface of the acetabulum and some were associated with osteolysis. The median serum cobalt and chromium levels were relatively constant over time, and were much higher than the detection level throughout the study period. The cobalt level was 1.5 μg/L 1 year after implantation, and 1.44 μg/L 9 years after implantation.

**Interpretation** Revisions for aseptic loosening and radiographic findings in the sockets led us to halt metal-on-metal-backed polyethylene cemented hip arthroplasty procedures. If the rigidity of the cemented socket is a reason for loosening, excessive release of metal ions and particles may be involved. Further investigations are required to confirm this hypothesis and to determine whether subluxation, microseparation, and hypersensitivity also play a role.

## Introduction

The second generation of metal-on-metal (MM) prostheses appeared in the late 1980s as a response to concerns about wear and complications associated with polyethylene, such as periprosthetic bone loss and aseptic loosening (Wroblewski et al. 1996). The second generation of MM prostheses for total hip arthroplasty (THA) had improved metallurgy, design, and precision in terms of the manufacture of the bearing surfaces (Weber 1996, Rieker et al. 2004). Early analysis of retrievals and wear simulation data indicated that the second generation of MM prostheses had minimal linear and volumetric wear after a run-in phase (Rieker et al. 2004). Short-term clinical results have also been encouraging (Weber 1996, Saito et al. 2006).

There are concerns about MM prostheses, because they generate chromium and cobalt ions and particles (Brodner et al. 2003, Rasquinha et al. 2006). In addition to problems related to tribology, questions remain about appropriate patient selection and about the medium- and long-term survival of cemented MM implants (Park et al. 2005, Catelas et al. 2006, Levai et al. 2006, Nich et al. 2006, Brown et al. 2007).

The purpose of this prospective study was to present data from the second generation of MM-backed polyethylene cemented hip prostheses at a mean follow-up time of 9 years. We conducted clinical and radiographic assessments as well as determination of serum cobalt, chromium, and titanium.

## Patients and methods

From January 1997 through December 2000, we prospectively followed a series of 113 consecutive patients at our institution who underwent primary total hip replacements (THA) using the Metasul Hip System (Zimmer, Warsaw, IN).

All patients provided informed written consent for this long-term clinical and biological prospective study. In accordance with the practice of the ethics board at our institution, they received complete information about the study, including the goals and the procedures for blood sampling.

Exclusion criteria were the presence of other orthopedic implants, a history of professional or dental exposure to titanium or cobalt, previous conservative surgical procedures on either hip, or renal disease. All enrolled patients were below 60 years of age at the time of surgery. In our practice, fewer than 20% of all patients undergoing primary THA during the study period were eligible for inclusion in this study.

Mean age at the time of arthroplasty was 54 (30–60) years. 53 females and 56 males were included. The etiologies were primary arthritis (121 cases) and osteonecrosis (13 cases). Mean body mass index was 26 (19–34). During the preoperative period, 77% of patients had an activity level graded as 4 or 5 according to the Devane activity score (Devane and Horne 1999). Clinical data for each patient were obtained by office interview and physical examination before surgery, and at each follow-up examination. Hip function results were rated using the Harris hip score grading system.

Of the 113 patients (138 hips), 4 patients (4 hips) were lost to follow-up in the first 5 years. At their last review, they had no pain and none had loosened implants.

The remaining 109 patients (134 hips) were available for complete clinical and radiographic analysis 7–11 years post-operatively. No patients in the series died.

### Implant characteristics

We used cemented Metasul cups (Zimmer, Warsaw, IN) for all replacements. This cup is composed of a single component, namely a 3-mm thick cobalt-chromium (Co-Cr) inlay embedded in a polyethylene socket. The cemented femoral stem (Alizé; Fournitures Hospitalières, Quimper, France) is collarless, oval in cross section, and straight. The implant was made of titanium alloy (Ti-6Al-4V) with a polished surface coated with titanium oxide (TiO_2_) obtained by anodization. 6 stem sizes were available. The modular stem was combined with a 28-mm Metasul femoral head (Zimmer) with a 12/14, 5°43’ taper.

Head-neck compatibility was assessed by a unitary control for the taper angle (precision: 1 minute) and by the diameter at the base and the diameter at the summit (precision: 1 micron) (Pexit Dorsey Gage, Cambridge, UK). Palacos Genta cement (Scherring Plough, Brussels, Belgium) was used for both components.

### Surgery

All procedures were performed following the standard practice at our institution, namely using an anterior approach on a Judet orthopedic table, by 4 different surgeons. To obtain a complete and thick cement mantle, the femoral canal was over-reamed by 2 mm and distally occluded by a resorbable femoral plug (Synplug; Zimmer). The cement was injected in retrograde fashion and pressurized by a hand-driven syringe.

### Radiographic assessment

Anteroposterior and lateral radiographs of each hip were taken before and immediately after surgery, 6 weeks after discharge from the hospital, and at 3 months, 6 months, 1 year, and then annually. Acetabular radiolucent lines and osteolysis were measured according to the zones described by DeLee and Charnley. Radiographic loosening of the cup was defined as the presence of radiolucent lines measuring 2 mm in at least two DeLee-Charnley zones, axial cup migration of > 5 mm, or > 5° of change in cup inclination on the anteroposterior radiographs of the pelvis.

On the femoral side, we assessed the presence and progression of radiolucent lines according to Gruen et al., calcar resorption or atrophy, subsidence, periprosthetic osteolysis, and cortical hypertrophy. Loosening of the stem was defined as migration exceeding 3 mm or a continuous radiolucent line wider than 2 mm. Radiographic measurement of wear was not possible as no distinction could be made between the edge of the femoral head and the metal articulation surface of the acetabular component.

### Serum ion determination

Titanium, cobalt, and chromium ion measurements were performed in all patients. None of the patients had renal insufficiency or chromium and cobalt devices of any kind that could create bias in the serum metal determinations. Blood samples were taken just before implantation and 3 months, 6 months, 1 year, and every year postoperatively until the last endpoint. To avoid metal contamination, blood samples were drawn using a sampling kit dedicated to trace element determination; this kit included an S-Monovette needle (ref. 85.1162.400; Sarstedt, Marnay, France) and lithium heparin in a 7.5-mL S-Monovette for trace metal analysis (ref. 01.1604.400, Sarstedt). All metal ion measurements were performed on 2 samples. Titanium was measured in diluted blood plasma by inductively coupled optical emission spectrometry (ICP-OES) on a JY24 spectrometer (Jobin Yvon, Longjumeau, France). The detection limit for titanium was 1.4 μg/L of plasma. Concentrations under the detection level were set at half the detection limit value (0.7 μg/L) to allow conventional statistical analysis. The serum cobalt and chromium levels were also determined in the same serum samples. Until 2004, these concentrations were simultaneously determined by electrothermal atomic absorption spectrometry using a 5100 spectrometer, and thereafter by using a SIMAA 6100 spectrometer (Perkin Elmer, Courtabœuf, France). In our laboratory, the detection limits of cobalt and chromium in serum were 0.3 μg/L. Concentrations below that level were defined as 0.15 μg/L to allow statistical analysis. As an internal quality control, Seronorm levels I and II (Sero, distributed by Ingen, Rungis, France) were also determined in each analytical session.

### Statistics

All statistical analyses were performed using SAS 9.1 software (SAS institute, Cary, NC). Prosthesis survival analysis was calculated using the Kaplan-Meier method with exchange of the cup, the stem, or both components as endpoint.

Our study included 25 bilateral joint replacements, and the events of these cannot be considered statistically independent. The statistical methods used required independent observations, however. Thus, we included only randomized observations from 1 hip of each patient in all statistical tests.

**Figure:**
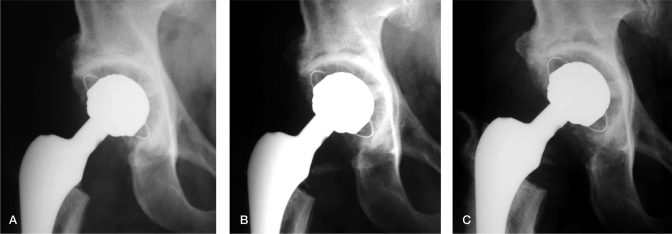
Progressive radiolucent lines at the bone-cement interface of the socket, leading to aseptic loosening at the 7-year follow-up. A. After one year. B. After 5 years. C. At the 7-year follow-up.

Serum metal levels were expressed as medians with twenty-fifth and seventy-fifth percentiles (IQR). Normally distributed data were analyzed with t-tests or ANOVA, and non-parametric data were analyzed with the Mann-Whitney U test.

Cups were categorized as either small (44–50 mm) or large (52–60 mm). Chi-square tests were performed to compare patients with and without radiolucent lines. Fisher’s exact tests were performed to compare patients with and without cup revision. Log-rank test was performed to determine a surgeon factor and whether the rate of loosening and revision was dependent upon surgeons. Statistical significance for all tests was set at p < 0.05.

## Results

### Clinical results

The mean Harris hip score improved from 39 (15–68) preoperatively to 91 (83–97) at the latest follow-up (p < 0.05).

### Survivorship

Considering revision for any cause as endpoint, the cumulative survival rate was 89% (95% CI: 80–94) at 9 years. The survivorship for cup revision due to aseptic loosening was 91% (95% CI: 82–96). With the endpoint as revision of the femur for aseptic loosening, the survivorship was 99% (95% CI: 93–100). The rates of revision and loosening were not significantly different for the different surgeons (p > 0.3).

### Complications

2 recurrent dislocations required revisions due to impingement between the titanium femoral neck and the edge of the Co-Cr insert of the cup. These dislocations occurred 1 month and 6 months after implantation. At the time of revision, the components were not loose; black staining of the joint space was noted, probably due to titanium release from femoral neck notches. In both cases, serum titanium levels were high due to release from the femoral neck lesions. All of the components were revised with the same polished cemented stem and a cemented polyethylene cup.

8 revisions were performed for loosened Metasul cemented cups with radiolucencies greater than 2 mm, axial migration, and osteolysis (Figure). 7 loosened cups were recorded in the unilateral group (84 hips) and the remaining one was in the bilateral group (50 hips). No relationship was detected between cup size and cup loosening (p = 0.70). Initially, all of the sockets had good cementing and no radiolucent lines. In 3 of the 8 cases, the THAs were revised using MM bearing surfaces (2 cases) or stainless steel-on-polyethylene bearing surfaces (1 case). Due to the serum levels of cobalt and the macroscopic metallosis observed at the time of revision, bearing surfaces were converted to ceramic-on-ceramic using a new cementless stem in 2 other revisions. In the last 3 cases, hips were revised using ceramic-on-ceramic heads with sleeves (Ceramtec, Plochingen, Germany) with conservation of the titanium femoral stems.

5 years after implantation, 1 case of progressive subsidence due to poor cement technique required revision; only the stem was changed.

1 prosthesis was revised due to persistent and unexplained pain; the implant was not loose. The serum cobalt level was more than 20-fold higher than the detection limit. At the time of revision, no abnormality was observed apart from massive and macroscopic metallosis in the joint. The bearing surfaces were changed for the ceramic-on-ceramic implants (the stem was not changed) and the symptoms disappeared.

### Radiographic results

Radiolucent lines and osteolysis around the cup were observed in 26% of the hips (35/134). They were all located at the bonecement interface, beginning always in zone 1—early and progressing with time. 5 of these 35 cups were considered loose due to there being radiolucent lines more than 2 mm and occupying at least 2 zones of DeLee et Charnley.

No relationship could be found between the occurrence of the radiolucent lines and cup size, cup and femoral position (cup inclination and anteversion, femoral anteversion, offset), body mass index, or Devane activity score.

3 stem subsidences were recorded as being due to poor cementing technique. 2 were inferior to 5 mm and slowly changed with time; these may require revision in the future. The third was revised as previously mentioned. It was associated with osteolysis in Gruen zone 6. Non-progressive femoral radiolucent lines were present in zone 1 at the cement-prosthesis interface in 10 hips, and had spread into zone 7 in 6 more hips. We did not observe femoral hypertrophic reaction around the distal stem, or calcar resorption.

### Serum levels of metals

In the 84 patients with unilateral MM hip replacements, the median serum cobalt level was relatively constant at 5- to more than 6-fold greater than the 0.3 μg/L detection limit (Table 1). Thus, serum cobalt levels did not increase over time, as found by Brodner et al. (2003). The median serum cobalt level was 1.4 μg/L at 1 year, 1.7 μg/L at 3 years (the highest median level), 1.3 μg/L at 5 years, and 1.6 μg/L at 9 years. The highest level of serum cobalt, 47 μg/L, was detected in 1 of the 2 patients with recurrent early dislocation and impingement between the titanium taper and the Co-Cr acetabular articulation. The highest level of serum titanium, 61 μg/L, was also detected in this patient. In the group of 8 patients whose hip prostheses were revised for aseptic loosening, the median serum cobalt level was 26 μg/L (0.95–34) just before surgery. The median serum titanium and chromium levels were also relatively constant (Table 1).

**Table 1.** Serum levels (median and IQR) of cobalt, chromium, and titanium (in μg/L) in the unilateral metal-on-metal hip replacements group

	1 year	3 years	5 years	7 years	9 years
No. of patients	84	84	84	84	56
Cobalt **^a^**	1.41 (1.04–2.78)	1.69 (1.04–2.46)	1.30 (1.01–2.01)	1.69 (1.12–3.28)	1.55 (1.05–2.79)
Chromium **^a^**	2.18 (1.49–3.37)	2.05 (0.94–3.21)	1.70 (0.90–3.41)	1.42 (0.80–2.25)	1.49 (0.72–2.0)
Titanium **^b^**	0.70 (0.70–0.70)	0.70 (070–0.81)	0.70 (0.70–070)	0.70 (0.70–1.96)	0.70 (0.70–1.61)

**^a^** In our laboratory, the detection limit of cobalt and chromium in serum was 0.30 μg/L. Concentrations below that limit were defined as 0.15 μg/L.

**^b^** The detection limit for titanium was 1.40 μg/L of plasma. Concentrations under the detection limit were set at half the detection limit value (0.70 μg/L).

In the 25 patients with bilateral MM hip replacements, the cobalt serum levels were always higher than in the unilateral group but only significant (p < 0.05) at 3 and 5 years. Regarding chromium, the values were also always higher than in the unilateral group but they were significant (p < 0.05) at 3, 5, and 7 years (Table 2).

**Table 2.** Serum levels (median and IQR) of cobalt, chromium, and titanium (in μg/L) in the bilateral metal-on-metal hip replacements group

	1 year	3 years	5 years	7 years	9 years
No. of patients	25	25	25	25	15
Cobalt	1.69 (1.13–4.66)	2.33 (1.66–4.15)	2.82 (1.65–4.69)	1.89 (1.35–3.65)	2.03 (1.09–4.82)
Chromium	2.60 (1.45–5.52)	2.61 (1.45–5.64)	2.83 (1.81–5.48)	2.41 (1.73–4.48)	2.99 (1.97–4.40)
Titanium	0.70 (0.70–0.85)	0.70 (0.70–1.62)	0.70 (0.70–0.80)	0.70 (0.70–2.16)	0.80 (0.70–1.83)

The median titanium concentration stayed within a constant range, and the median value was always below the detection limit of 1.40 μg/L (Table 1). In contrast, patients whose implants had failed stems had the highest titanium serum levels in the series at the time of failure; these levels ranged from 9.1 to 60 μg/L and were much higher than the detection level. For patients with loosened cups, the titanium serum levels remained below the limit of detection.

In the bilateral group, titanium serum levels were also constant and always below the detection limit.

## Discussion

Our findings in this study of MM-backed polyethylene cemented hip arthroplasties with 28-mm femoral heads raise serious concerns. The survivorship at 9 years with revision due to aseptic cup loosening was 91%. In a study with 10-year follow-up based on the Swedish Hip Arthroplasty register (Malchau et al. 2002), the survivorship was 93% for any revision with cemented metal-on-polyethylene. We observed early and progressive radiolucent lines and osteolysis in 26% of the cups, which raises questions about their durability. At the endpoint of this study, 5 additional cups were considered loosened and required revision.

With the use of cemented MM arthroplasties, radiolucent lines have been described in the literature during the early postoperative period. However, their long-term evolution has remained unclear (Weber 1996, Levai et al. 2006, Nich et al. 2006). In a study of cemented Metasul cups, Levai et al. (2006) reported a high frequency of acetabular radiolucent lines; 5-year survivorship using surgical revision as the endpoint for aseptic cup loosening was 94%. The thickness of polyethylene has been suggested as an explanation for these results (Levai et al. 2006, Nich et al. 2006). Thinner polyethylene may lead to increased socket rigidity, resulting in greater constraints at the bone-cement interface; indeed, this has been observed with other types of rigid cemented cups (Nich et al. 2003).

We found no relationship between the occurrence of the acetabular radiolucencies or revisions and cup size or implant position. Moreover, osteolysis was found to be associated with progressive radiolucencies, as already reported for other second-generation MM implants, including cementless implants (Park et al. 2005, Grubl et al. 2007). There may be additional factors to explain the rate of aseptic loosening, but metal debris from the implants most likely contributes to wear and corrosion (Yan et al. 2006).

Acute dislocation and joint laxity with subluxations and microseparations can induce local and systemic release of chromium and cobalt particles (Williams et al. 2006, Brown et al. 2007). Simulator studies have shown that there is a substantial release of particles when microseparation is introduced into the gait cycle (Williams et al. 2006). Our analysis of retrieved femoral heads and liners showed lesions typical of microseparation with stripe wear. Mapping of the stripe wear lesions to balls and to cup rims has indicated that this is an edge-contact phenomenon.

The surfaces of MM bearings release Co-Cr wear particles that are not biologically inert (Willert et al. 1996). In vitro studies have shown production of the bone-resorbing cytokines TNF-α and IL-1 by cells cultured in the presence of Co-Cr particles (Catelas et al. 2004). Light microscopy has shown that small metal wear particles can aggregate into larger bioactive particles and that they can induce osteolysis and aseptic loosening (Catelas et al. 2004, Brown et al. 2007). In addition to the possible adverse effects of Co-Cr particles derived from the bearing surface, the effects of titanium particles should also be considered (Haynes et al. 1993). We used cemented femoral titanium (Ti–6Al–4V) stems in combination with the MM bearing surface, which is a controversial choice. It has been suggested that cemented titanium stems may result in micromotion, corrosion, and production of metal debris, leading to aseptic loosening (Scholl et al. 2000). In addition, titanium debris may be generated at the metal head/neck modular junction. We did not find any evidence to support these hypotheses, however. First of all, the performance of the stem was excellent, with 99% survivorship at the last review; these results are in accordance with the results of previously published studies (Nich et al. 2003, Boyer et al. 2008). The only elevated serum titanium levels were the result of stem loosening due to poor cementing technique or to neck impingement (in the 2 cases of dislocation). In all other patients, serum titanium levels were always below the limit of detection (1.4 μg/L), even for patients with revisions due to cup loosening. In these cases, peroperative examination of the taper did not reveal any significant corrosion, fracturing, or crevicing.

Particles released from the implants can also corrode in the biological fluid environment and generate metal ions (Bhamra and Case 2006). Ions released from MM THAs have been detected by analysis of serum samples, whole blood, and erythrocytes (Brodner et al. 2003, Rasquinha et al. 2006). Most of these studies found cobalt concentrations around 1.0–1.5 μg/L that remained constant. In addition, the cobalt levels were 8- to 10-fold higher than the detection limit and did not reflect the changes in wear, which were described as involving 2 successive phases (Brodner et al. 2003). Our results, at a mean of 9-years follow-up, were in agreement with those of earlier studies: median serum cobalt levels were 1.41 μg/L after 1 year and 1.55 μg/L at the endpoint of the study.

Metal ions released from implants can activate the immune system by forming metal-protein complexes, which can in turn induce implant-related hypersensitivity reactions with delayed cell-mediated responses (Hallab et al. 2001). Park et al. (2005) described 9 patients with early osteolysis of the greater trochanter after cementless metal-on-metal hip replacement. Their data supported a role for hypersensitivity, as histological analysis revealed periprosthetic tissue infiltration of CD3- positive T-cells and CD68-positive macrophages; immunohistochemical analysis showed cytokines IL-1 and TNF-α.

In conclusion, the results of this prospective series of 28- mm MM-backed polyethylene cemented hip arthroplasties were disappointing, and these implants were inferior to metal-on-polyethylene implants with the same femoral head diameter (Malchau et al. 2002). Constraints at the cement-bone interface may be involved due to the sandwich structure of cemented MM cups (Nich et al. 2003). Titanium stems do not appear to have played a role in implant failure. The release of metal particles and ions from the bearing surfaces may also have ramifications, such as induction of delayed reaction against the implants. Because of the high rate of acetabular loosening (with 91% survivorship at 9 years), we no longer use bearings with metal-on-metal surfaces.
